# Raman and Conductivity Analysis of Graphene for Biomedical Applications

**DOI:** 10.3390/ma9110897

**Published:** 2016-11-04

**Authors:** Chao Qiu, Kevin E. Bennet, Tamanna Khan, John D. Ciubuc, Felicia S. Manciu

**Affiliations:** 1 Department of Physics, University of Texas at El Paso, El Paso, TX 79968, USA; cqiu2@utep.edu (C.Q.); ttkhan@miners.utep.edu (T.K.); jdciubuc@miners.utep.edu (J.D.C.); 2 Division of Engineering, Department of Neurologic Surgery, Mayo Clinic, Rochester, MN 55905, USA; Bennet.Kevin@mayo.edu; 3 Border Biomedical Research Center, University of Texas at El Paso, El Paso, TX 79968, USA

**Keywords:** biomaterials, confocal Raman mapping, Drude model, conductivity, infrared absorption, dopamine detection

## Abstract

In this study, we present a comprehensive investigation of graphene’s optical and conductive properties using confocal Raman and a Drude model. A comparative analysis between experimental findings and theoretical predictions of the material’s changes and improvements as it transitioned from three-dimensional graphite is also presented and discussed. Besides spectral recording by Raman, which reveals whether there is a single, a few, or multi-layers of graphene, the confocal Raman mapping allows for distinction of such domains and a direct visualization of material inhomogeneity. Drude model employment in the analysis of the far-infrared transmittance measurements demonstrates a distinct increase of the material’s conductivity with dimensionality reduction. Other particularly important material characteristics, including carrier concentration and time constant, were also determined using this model and presented here. Furthermore, the detection of micromolar concentration of dopamine on graphene surfaces not only proves that the Raman technique facilitates ultrasensitive chemical detection of analytes, besides offering high information content about the biomaterial under study, but also that carbon-based materials are biocompatible and favorable micro-environments for such detection. Such information is valuable for the development of bio-medical sensors, which is the main application envisioned for this analysis.

## 1. Introduction

Since being discovered, graphene [1] has attracted significant research interests due to its exceptional and unique properties, such as high carrier mobility at room temperature, transparency, and conductivity. Consequently, it has been investigated as a feasible candidate for use in device fabrication in numerous areas: electronics [2,3,4,5,6,7,8,9,10], optics [11,12], photonics [3,13], micro/nano-mechanics [14,15,16], and, recently, biomedical engineering [17,18,19,20]. As a two-dimensional material, graphene shows very good thermal and electrical conductivities, which, combined with its unique optical properties, make it suitable for a variety of applications [1,2,3,4,5,6,7,8,9,10,11,12,13,14,15,16,17,18,19,20,21,22]. Many efforts have been made in the last decade related to the conductivity of graphene—in finding accurate measuring methods, as well as in fabricating new graphene-based materials with improved performance [23,24,25]. In this context, modification of graphene has become a trend. For example, the integration of metal nanowires in graphene thin films has led to reduced resistance of the films [23,24]. An increase in solar cell efficiency due to improvement of the direct-current to optical conductivity ratio has also been reported [24]. Excellent optical and electric performance has been achieved with core-shell nanowires based on Cu and reduced graphene oxide [25]. Furthermore, it has been demonstrated that chemically modified graphene can be embedded in polymeric matrices, too, for development of electrically conductive and flexible composite materials for energy, environmental, and medical application development [17,18,19,20,26,27,28,29]. However, production of large-area and high-quality graphene films on desired substrates or perfect, homogeneous embedding into polymeric matrices is necessary if device fabrication is envisioned. To overcome this challenge, different methods of graphene production have been proposed and developed, such as standard exfoliation [1], chemical vapor deposition [8,15], and chemical synthesis [2,28,29].

Parallel with efforts in creating new materials, there has been a continuous interest in studying fundamental properties of graphene [21,30]. In this context, Raman spectroscopy has traditionally played a key role in structural characterization of carbon-based materials. Not only has this technique been widely used in identifying the main fingerprints of graphitic materials such as the D, G, and 2D bands, but also in determining the number of layers, structural changes and defects introduced by layer folding, and interlayer shear modes [21,30]. Therefore, as a first step, we present in this work a detailed confocal Raman microscopic investigation that combines analysis of Raman spectra with direct visualization of graphene domains consisting of either single layers, a few layers, or multiple layers.

We also present and discuss an alternative method to study the conductivity of graphene using the Drude model. This model has been successfully applied to such an analysis for metallic thin films [31,32] and, very recently, for carbon based materials [33,34,35,36]. Two types of optical transitions (e.g., intraband and interband) are known to contribute to absorption in graphene. Considering their dependence on the spectral range of interest, the examination of the free carrier response of graphene in the far-infrared (IR), at frequencies equivalent to the terahertz regime, provides understanding of the intraband transitions. The far-IR is also the spectral region associated with Drude model applicability. It is also important for development of IR tunable optoelectronic devices [33,34]. Absorption that is nearly frequency independent has been reported for single, a few, and multi-layers of graphene in the mid- and near- infrared regions, which are dominated by interband transitions [37].

It is worth noting here that the majority of such articles report the conductive response of graphene at different applied voltages and temperatures. In this work, we consider a comparative analysis between the known conductivity of graphite and that of graphene, at normal ambient conditions and under no voltage application. Thus, the observed trend in increased conductivity is due to the confinement and reduced dimensionality that affects the materials’ properties. Any subsequently applied voltage is expected to boost this conductivity, up to a limit which would be dependent on the number of carriers and the mean free time between their collisions with the lattice ions and among themselves.

Finally, but very important to our envisioned applications, we demonstrate that, indeed, analytes such as dopamine (DA) in this work, can be detected using graphene, providing direct evidence of its applicability for future biosensor development. Carbon-based materials are not only known to be favorable micro-environments for facilitating direct electron transfer, and, consequently, redox reactions for DA, but also for biocompatibility [17,18,19,20,38,39,40]. Thus, these two properties of graphene, conductivity and biocompatibility, make it an ideal coating electrode material for fast-scan cyclic voltammetry (FSCV), a recording method employed in deep brain stimulation (DBS) and a state-of-the-art neurosurgical intervention technique.

## 2. Materials and Methods

### 2.1. Materials and Equipment

Whether graphene consisting of just a few layers or of a greater number of layers was to be prepared, a mechanical exfoliation method was applied following previous reports [1,40]. Generally, to obtain multi-layer graphene, a highly oriented pyrolitic graphite (HOPG) crystal was exfoliated several times with Scotch^®^ tape. Then, the graphene flakes were transferred to the Si substrate. For either a few-layers or a single-layer of graphene, a modified mechanical exfoliation method was used, which is largely similar to that employed by Huang et al. [40]. In our case, instead of immediately removing the graphite-loaded tape from the substrate, we first annealed it with water vapor for 3–5 min. Then, the tape was left to naturally cool down to room temperature and removed. Thus, by increasing the force and the contact area between the Si and graphite, improved quality of the resulting graphene flakes was achieved with this vapor-assisted exfoliation method. The outcome was the successful production of just a few-layers, as well as single-layer graphene on the Si substrate. Huang et al. [40] directly annealed the graphite-loaded tape on the Si/SiO_2_ substrate on a hot plate for 2–5 min at approximately 100 °C. However, we found that following their procedure also results in additional residue on the substrate from the tape’s glue. These unwanted impurities were avoided by the vapor-assisted exfoliation method used here.

The confocal Raman study utilized an Alpha 300R WITec system (Ulm, Germany) equipped with a 532 nm neodymium-doped yttrium aluminium garnet (Nd:YAG) laser and a back-illuminated CCD camera. A 100X objective with a numerical aperture of 0.9 was used for these measurements. To obtain Raman mapping images, selected areas of the sample were raster scanned under the laser beam and arrays of the Raman spectra were recorded with an acquisition time of 50 milliseconds for each pixel. The spectra were then mathematically processed for analysis of the intensity distribution of the Raman peaks of interest. The WITec Control software (Ulm, Germany) was utilized for controlling the piezo stage during the scans as well as for data acquisition. The Cluster Analysis Tool of the WITec Project Plus software was employed for further data analysis.

The far-IR transmission measurements were performed under vacuum using a Bruker IFS 66v FT-IR spectrometer (Billerica, MA, USA) with a Ge-coated Mylar beam splitter and deuterated triglycine sulfate (DTGS) detector. For each spectrum, 256 scans were acquired and the resolution was 4 cm^−1^.

### 2.2. Drude Theoretical Approach

The ratio, T(ω), of the transmission of radiation of angular frequency ω through a thin conductive film deposited on a substrate, T_C_(ω), to the transmission through the substrate alone, T_S_(ω), without consideration of multiple reflections is given by [31,32]:

(1) $$T\left(\omega \right)\equiv \frac{T_{C}\left(\omega \right)}{T_{S}\left(\omega \right)}=\frac{1}{{\left|1+\frac{\sigma \left(\omega \right)Z_{0}d}{n_{S}+1}\right|}^{2}}$$

where σ(ω) is the conductivity, Z_0_ = 377 Ω is the impedance of free space, d is the thickness of the thin film, and n_S_ is the index of refraction of the substrate (in this case, for Si, n_s_ = 3.4). The thicknesses of the samples used were about 1 µm for HOPG, 9.4 nm for multi-layer graphene, and 1.7 nm for the sample containing a mixture of single-layer and few-layer graphene.

In the classical approach, the expression of conductivity from a Drude free electron model in the presence of scattering is: [28,29]:

(2) $$\sigma \left(\omega \right)=\frac{\sigma_{0}}{1+i\;\omega \;\tau }$$

where σ_0_ = (*ne*^2^τ)/m* is the Drude dc conductivity, *n* is the electron density, *e* and m* are the electron charge and effective mass, respectively, and τ is the mean free time between collisions of the carriers with the lattice ions. Inserting (2) in (1) followed by mathematical manipulations that were explained elsewhere [35], gives:

(3) $$\frac{T\left(\omega \right)}{1-T\left(\omega \right)}=a+b\omega^{2}$$

The fitted parameters *a* and *b*, obtained from experimental data, provide:

(4a) $$\tau =\sqrt{\frac{b}{a}}$$

(4b) $$\sigma_{0}=\frac{n_{S}+1}{Z_{0}d}\left(\sqrt{1+\frac{1}{a}}-1\right)$$

(4c) $$n=\frac{m^{*}\left(n_{S}+1\right)}{Z_{0}de^{2}}\left(\sqrt{1+\frac{1}{a}}-1\right)\sqrt{\frac{a}{b}}$$

Although the electrons in graphene behave as massless Dirac fermions [33], for consistency in the theoretical treatment of the samples analyzed here (e.g., from graphite to a few graphene layers, all with measurable thicknesses), we still consider a classical approach for the Drude model, instead of a quantum approach, the latter being more applicable for just a single-layer of graphene. However, a majority of literature sources still report defects and nonuniform coverage of the substrate; only locally was single-layer graphene observed. Even though better quality graphene is attainable using chemical vapor deposition (CVD) as the growth method, there is still a high possibility of creating defects in the material, mainly arising from the subsequent necessary transfer of graphene to the desired substrate, or from embedding it into polymer matrices.

## 3. Results and Discussion

**Figure 1:**
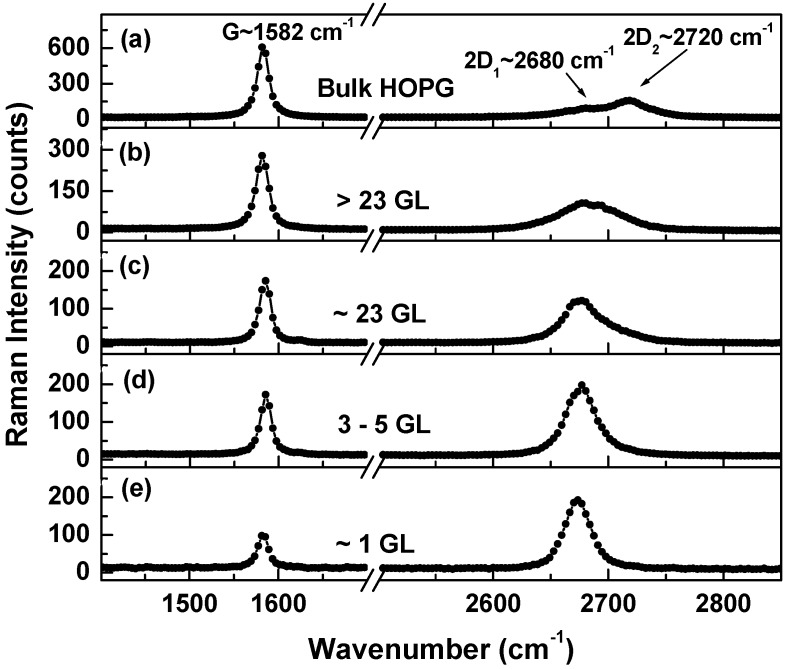
(**a**–**e**) Raman spectra recorded in different spots showing the behavior of the G and 2D bands for a single-layer, a few-layers, and multi-layer graphene. The Raman spectrum of bulk HOPG is also presented for comparison.

The Raman spectra that are shown in Figure 1a–e were recorded at different spots on the samples. They demonstrate an intrinsic inhomogeneity in each of these samples regarding the numbers of graphene layers. For comparison, we also incorporate in Figure 1a the spectrum of bulk HOPG.

While both bands, the 2D_1_ at ~2680 cm^−1^ and the 2D_2_ at ~2720 cm^−1^ can be observed in the Raman spectrum of the bulk HOPG, just a single feature centered at 2675 cm^−1^ and with a full width at half maximum (FWHM) of ~28 cm^−1^ is seen in Figure 1e of single-layer graphene. There is an expected decrease in the intensity ratio of G (at 1582 cm^−1^) to 2D peaks, I_G_/I_2D_, with the decrease in the number of graphene layers. Similar results have been previously reported in the literature [21]. Our tentative assignments for the number of layers in Figure 1b–e have been based on this I_G_/I_2D_ ratio, which varies from 0.28 for a single-layer to more than 1.4 for multi-layers. Besides an increase in the broadness of the 2D (i.e., G’) band, an evolution in the shape of this feature is observed with the increase in the number of graphene layers. As well explained by Malard et al. [21], a concept essential to understanding the evolution of this shape is the phonon dispersion of graphene. The 2D band is known to originate from a second-order Raman scattering process in graphene. However, its shape evolves in multi-layer graphene with the number of Raman peaks and transitions allowed by resonance processes involving optical phonons near the K point (or Dirac point). The combinations of these modes ultimately result in the splitting of the 2D_1_ and 2D_2_ bands mentioned above. In the case of a single graphene layer, the remarkable intensity of the 2D band relative to that of the G band can be understood in terms of a triple resonance process. A not so dramatic change in shape and broadness is expected for the G band at 1582 cm^−1^, which is associated with the doubly degenerate phonon mode of E_2g_ symmetry at the Brillouin zone center Г. It is also the single band originating from a normal first order Raman scattering process in graphene.

**Figure 2:**
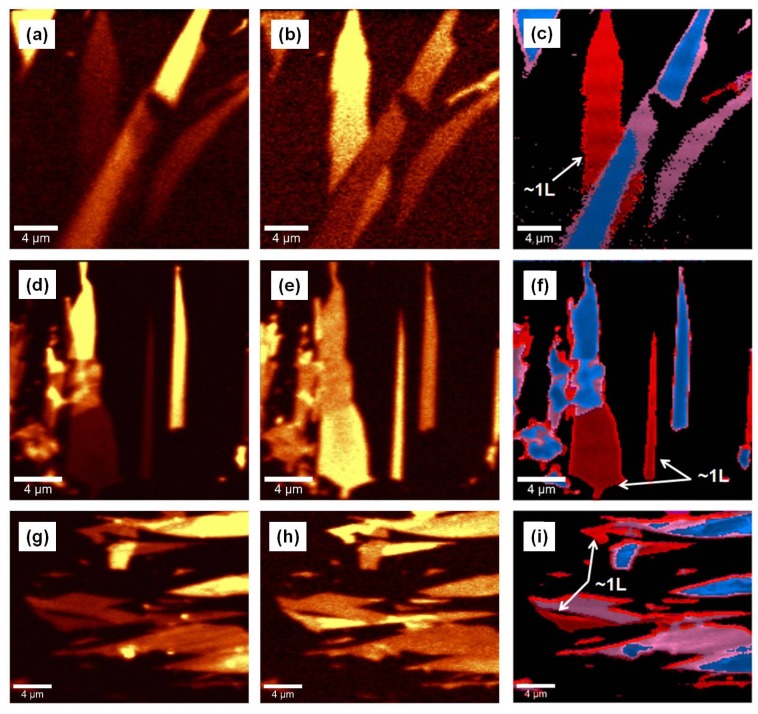
Confocal Raman mapping images screening: (**a**,**d**,**g**) the location of the G band; and (**b**,**e**,**h**) the location of the 2D band. Yellow pseudo-color in the images corresponds to higher intensity of the filtered Raman feature; (**c**,**f**,**i**) Images preformed with Cluster Analysis software (Ulm, Germany) revealing a mixture of a single-layer and a few-layers of graphene. The arrows mark the presence of a single-layer of graphene in the samples.

One can directly visualize the intensity changes between the G and 2D bands in Figure 2a–i, where the confocal Raman images of these bands are presented.

The images were produced by selectively filtering the location of the aforementioned features. Bright yellow represents higher intensity of the selected band. An expected complementary effect is depicted in these images, where the visibility of high intensity regions in images (a), (d), and (g), which are representative of G band locations, can be compared with that of the correspondingly low intensity in the (b), (e), and (h) images. Similarly, the high intensity 2D band locations in the (b), (e), and (h) images exhibit a low intensity in the (a), (d), and (g) images. For further easier visualization of the combined effect, the Cluster Analysis software (an unsupervised method that clusters data by their similarity) has been employed in the corresponding images presented in Figure 2c,f,i. A good match between the single-layer regions which are pseudo-colored with red and marked with arrows in these latter images and the high intensity regions associated with the 2D band is observed (e.g., bright yellow regions in Figure 2b,e,h). Thus, confocal Raman mapping facilitates better and more accurate detection of single-layer regions, which would be otherwise very hard to notice due to the known transparency of graphene. This impediment has usually been overcome by using Si substrates covered with SiO_2_ thin film insulators [4,21,40]. The presence of bilayers and trilayers of graphene (soft purple pseudo-color), as well as of a few graphene layers (blue pseudo-color) is also detected in Figure 2c,f,i.

**Figure 3:**
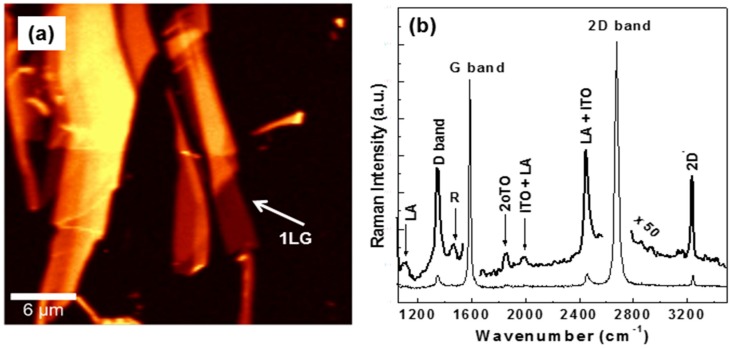
(**a**) Confocal Raman mapping of the G band depicting a microtube-like structure or of graphene whiskers; and (**b**) the associated Raman spectrum of image (**a**). New weak Raman vibrations, as labeled, are detected besides the characteristic graphene features.

Furthermore, as depicted in Figure 3a, potential curling of a single-layer of graphene into a microtube-like structure or whiskers could happen, too. Since, in this case, no remarkable difference between the Raman images of the G and 2D bands was observed, just the former is shown. The arrow again indicates the presence of an uncurled single-layer of graphene. The effect of rolling up the graphene sheet on the phonon modes is detectable in Figure 3b, where the Raman spectrum associated with this image is presented. Although very weak, there are new Raman vibrations at ~1100 cm^−1^ (LA mode), 1345 cm^−1^ (D band), 1505 cm^−1^ (R band), 1862 cm^−1^ (2oTO), 1985 cm^−1^ (iTO + LA near Г), 2452 cm^−1^ (LA + iTO near K), and 3240 cm^−1^ (2D’) in the enhanced spectrum [41,42,43]. A defect in the structure of graphene is needed to activate the D band, which is due to the breathing modes of six-atom rings and comes from the TO phonons around the Brillouin zone center K. It could also become active under double resonance that happens as an intervalley process, where two points belonging to the same cone are connected (i.e., K or K’). While the D and D’ peaks involve defects in the graphene structure, there is no such requirement for their overtones, the 2D and 2D’ peaks, respectively, as momentum conservation is satisfied [41,42]. The small R peak, which originates from the twist-induced wave vector in the superlatice, is a direct signature of folding that can occur accidentally during exfoliation [30,43]. Comparison of current data with those previously reported by Cong and Yu [43], suggests a medium to relatively large angle of rotation (i.e., approximately 12° < θ <20°).

**Figure 4:**
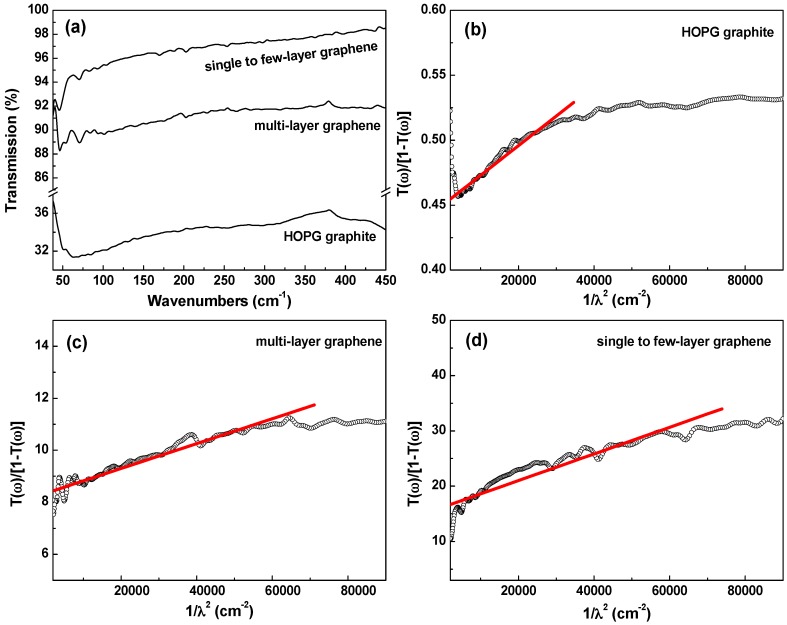
(**a**) Far-IR transmission results of three representative samples, as labeled; (**b**–**d**) Representative fits demonstrating linear dependence of the ratio of transmittance, T(ω), to [1 − T(ω)] as a function of 1/λ^2^ for the samples in Figure (**a**).

Even though many efforts have been employed in understanding the differences between theoretically predicted conductive properties of graphene and experimentally reported ones, there is still an unsettled argument. Multiple reasons contribute to these differences, such as those arising from sample to sample variation and intrinsic inhomogeneity (e.g., numbers of layers, lateral dimensions, and surface coverage), as well as from methods of fabrication. Since the samples analyzed here are definitely inhomogeneous, investigation of their conductivity and comparison with previously reported data would be of interest. Therefore, we present in Figure 4a the experimental transmission measurements of three representative samples, namely that of the bulk HOPG, of the multi-layer graphene, and of the single to few-layer graphene. To consider only the transmission of the conductive thin film, appropriate ratios to the transmission of the Si substrate were performed for all the graphs. Hence, the overall transmissions seen in these spectra are inversely related to the thicknesses of the films, which vary from 1 µm for HOPG to about 1.7 nm for samples containing a mixture of single-layer and few-layer graphene. The thickness of the multi-layer sample was about 9.4 nm.

There is relatively good agreement between the observed asymptotic absorption of ~2.3% (~97.7% transmission) and that predicted for a single-layer of graphene [1,36,37], despite the inhomogeneity of this sample as inferred by the above Raman data. An increase in this sample’s absorption to 8% is also seen at very low frequencies. At the other extreme is the strong absorption of the HOPG sample, which ranges from about 70% at wavenumbers below 250 cm^−1^ to about 65%. The multi-layer graphene sample has a relatively small variation in its absorption, from about 9% to 12%.

With confinement, broad features of around 34 and 75 cm^−1^ are observed in the spectra of single-layer and few-layer graphene and of multi-layer graphene samples. We assign these absorptions to the acoustic E_u_ mode and the breathing mode, respectively [44]. The lower frequency mode at ~34 cm^−1^ can also be atributed to the shear mode, which becomes IR active for more than two layers of graphene [44]. Unfortunately, due to the cutoff limit of our bandpass filter, we were not able to detect the shear (i.e., low-energy E_2g_ mode) and the breathing modes in our Raman measurements.

Figure 4b–d shows the derived ratios of the transmittance, T(ω), to [1 − T(ω)], as a function of 1/*λ*^2^ for the samples presented in Figure 4a. Extraction of the fitting parameters *a* and *b* (i.e., the intercept and the slope) is obtained from these graphs and further used for the calculation of sample conductivity, resistivity, time constant, and number of carriers, as described above. In this way, a prospective trend for these important material charteristics could be achievable. While the deviations from linear fits at lower wavenumbers (<30 cm^−1^) seen in the graphs are due to the detector cutoff limit, at higher frequencies, they are due to the characteristic Drude falloff limit. Furthermore, because of variation in the number of layers and, implicitly, of sample thickness, there is a variation from sample to sample in the suitable linear fit range, too.

A conductivity of 92 (Ω·cm)^−1^ and a resistivity of 11 × 10^−3^ (Ω·cm) were derived using the Drude model for the HOPG graphite sample. These values are in good agreement with those previously reported for graphite (i.e., a resistivity of 3−60 × 10^−5^ (Ω·m)) [45]. An increase in conductivity to 407 (Ω·cm)^−1^ and to 2138 (Ω·cm)^−1^ is obtained with dimensionality reduction, for the multi-layer graphene sample and the sample containing a mixture of single-layer to few-layer graphene, respectively. The current results for conductivity are slightly higher, but comparable with those reported in the literature for multi-layer graphene [27]. Behavior opposite to that of conductivity is observed for resistivity, which shows a decrease from 11 × 10^−3^ (Ω·cm) for the HOPG sample, to 2.5 × 10^−3^ (Ω·cm) for the multi-layer graphene sample, to, finally, 0.47 × 10^−3^ (Ω·cm) for the sample with a single-layer to several layers of graphene. An increase by an order of magnitude is also obtained for the number of carriers, *n*, with values of 3.58 × 10^18^/cm^3^ for HOPG, to 14.9 × 10^18^/cm^3^ for multi-layer graphene, and 46.3 × 10^18^/cm^3^ for graphene with a single-layer to several layers. There is almost no variation in the time constant (the inverse of the average time between two carrier-core collisions), with values ranging from 7.4 × 10^−14^ s, to 7.9 × 10^−14^ s, and to 13.4 × 10^−14^ s from the graphite sample to the graphene samples, respectively. These results demonstrate that, indeed, the intralayer conductivity is the dominant process.

**Figure 5:**
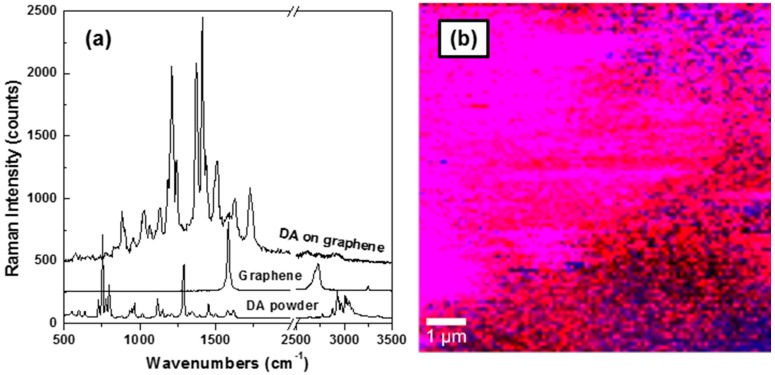
(**a**) Representative Raman spectra of standard dopamine powder, graphene and dopamine detected on a graphene surface, as labeled; (**b**) Confocal Raman mapping of dopamine (**red**) and graphene (**blue**). Magenta color is a combination of red and blue indicating the presence of both materials.

Analytes such as dopamine (DA) have been mainly detected using voltammetry as previously reported in the literature [17,18,19]. The targeted application in this work is a potential use of graphene for future biosensor development, and we show in Figure 5a,b that detection of such analytes is achievable by this means without voltammetry. For our study, graphene flakes were immersed overnight in a solution of 1.0 × 10^−6^ M of DA (standard powder purchased from Sigma Aldrich Chemicals Co., St. Luis, MO, USA). Besides the standard spectrum of powder DA, representative spectra of graphene and DA detected on a graphene surface are also presented in Figure 5a. Even though the laser power for DA detection on the graphene surface was reduced by at least 10 times, to about 100 µW, compared to that of the standard DA powder recording, which was performed with about 1 mW of laser power, an overall increase in the signal is depicted for the former spectrum, which was recorded for just 200 milliseconds. This observation suggests a graphene-enhanced Raman spectroscopy (GERS) effect for dopamine detection [46,47]. While much higher enhancement factors (EF) could be achieved by surface-enhanced Raman spectroscopy (SERS) (up to 10^10^ times), EF up to 10^2^ times by GERS have been reported [46]. The main difference in the EF obtained from the two spectroscopic methods arises from the method’s intrinsic mechanism. While SERS is based on an electromagnetic mechanism and noble metal substrates, a chemical mechanism is the underlying process of GERS. Not only does GERS have the advantages of repeatability and stability of the enhanced Raman signal, but it can also be used as a molecularly selective detection method, as higher EF are expected for analytes with the highest occupied molecular orbital (HOMO) and lowest unoccupied molecular orbital (LUMO) in the vicinity of graphene’s Fermi level [46]. Thus, with HOMO/LUMO energy levels of approximately −6.7 eV/−3.5 eV [48], dopamine is a viable candidate. A charge transfer process between the analyte and substrate could also contribute to this signal enhancement.

There are obvious shifts between the vibrational lines of DA powder and those of the analyte detected on the graphene surface in addition to the visible increases in the intensities of vibrational lines around 1220, 1370, 1450, 1510, 1620, and 1740 cm^−1^ in the spectrum of DA recorded on graphene. These observations imply interaction between DA and graphene. While disappearance of the low-frequency standard DA vibrations around 750 cm^−1^, which are associated with H–C–N bonds, indicate potential adsorption of DA molecules on the graphene surface, the vibrations around 1220, 1370, and 1450 cm^−1^ indicate deprotonation of DA molecules and changes in the stretching of the catechol carbon–oxygen stretching C–O bonds [49]. The 1620 and 1740 cm^−1^ peaks could arise from changes of the stretching vibration of C=C and the stretching vibration of conjugated carbonyl groups (-C=O) or of carboxyl groups (–COOH) on the edges of the basal planes, respectively. These latter peaks also suggest potential polymerization of DA on the graphene surface [50].

The visual detection of DA on the graphene surface by confocal Raman mapping is presented in Figure 5b. This image represents a combination of graphene locations (pseudo-colored with blue) with those of DA (pseudo-colored with red); the observed magenta color results from overlapping of blue and red. Although there is an expected non-uniform adsorption of DA on the graphene surface [20], all these results make it obvious that the Raman technique facilitates ultrasensitive chemical detection of analytes, besides offering high information content about the biomaterial under study.

## 4. Conclusions

In this study, we present detailed investigations by confocal Raman and Drude model analysis of changes and improvements in a material’s properties, as it transitions from 3D graphite to 2D graphene. Besides spectral recording by Raman technique, which shows whether there is a single-layer, a few-layers, or multi-layers of graphene, the confocal Raman mapping allows for distinction of such domains and a direct visualization of material inhomogeneity. Moreover, the far-IR transmittance measurements and use of the Drude-like model, both related to material electrical conductivity, demonstrate a distinct increase of its conductivity with dimensionality reduction, from 92 (Ω·cm)^−1^ for a HOPG sample to 2138 (Ω·cm)^−1^ for a sample with a single-layer to several layers of graphene. These measurements are also particularly suited to determining other important material characteristics, including carrier concentration that increase from 3.58 × 10^18^/cm^3^ for HOPG, to 14.9 × 10^18^/cm^3^ for multi-layer graphene, to 46.3 × 10^18^/cm^3^ for a sample with a single-layer to several layers of graphene. These latter results demonstrate that the intralayer transport is the dominant process that contributes to the material’s conductivity. Such information is valuable for development of bio-medical sensors, which is the main application envisioned for this current work. In this context, detection of dopamine on a graphene surface by Raman spectroscopy and microscopy is also presented and discussed. Thus, this study is significant not only for advancing the understanding of plasmon oscillations in graphene and their response in the IR region, but also for further design of graphene-based electronics and sensitive optoelectronics devices.
